# Cold-Adapted Influenza and Recombinant Adenovirus Vaccines Induce Cross-Protective Immunity against pH1N1 Challenge in Mice

**DOI:** 10.1371/journal.pone.0021937

**Published:** 2011-07-15

**Authors:** Mark R. Soboleski, Jon D. Gabbard, Graeme E. Price, Julia A. Misplon, Chia-Yun Lo, Daniel R. Perez, Jianqiang Ye, S. Mark Tompkins, Suzanne L. Epstein

**Affiliations:** 1 Division of Cellular and Gene Therapies, Center for Biologics Evaluation and Research, Food and Drug Administration, Bethesda, Maryland, United States of America; 2 Department of Infectious Diseases, University of Georgia, Athens, Georgia, United States of America; 3 Department of Veterinary Medicine, University of Maryland, College Park, Maryland, United States of America; University of Rochester School of Medicine, United States of America

## Abstract

**Background:**

The rapid spread of the 2009 H1N1 pandemic influenza virus (pH1N1) highlighted problems associated with relying on strain-matched vaccines. A lengthy process of strain identification, manufacture, and testing is required for current strain-matched vaccines and delays vaccine availability. Vaccines inducing immunity to conserved viral proteins could be manufactured and tested in advance and provide cross-protection against novel influenza viruses until strain-matched vaccines became available. Here we test two prototype vaccines for cross-protection against the recent pandemic virus.

**Methodology/Principal Findings:**

BALB/c and C57BL/6 mice were intranasally immunized with a single dose of cold-adapted (*ca*) influenza viruses from 1977 or recombinant adenoviruses (rAd) expressing 1934 nucleoprotein (NP) and consensus matrix 2 (M2) (NP+M2-rAd). Antibodies against the M2 ectodomain (M2e) were seen in NP+M2-rAd immunized BALB/c but not C57BL/6 mice, and cross-reacted with pH1N1 M2e. The *ca*-immunized mice did not develop antibodies against M2e. Despite sequence differences between vaccine and challenge virus NP and M2e epitopes, extensive cross-reactivity of lung T cells with pH1N1 peptides was detected following immunization. Both *ca* and NP+M2-rAd immunization protected BALB/c and C57BL/6 mice against challenge with a mouse-adapted pH1N1 virus.

**Conclusion/Significance:**

Cross-protective vaccines such as NP+M2-rAd and *ca* virus are effective against pH1N1 challenge within 3 weeks of immunization. Protection was not dependent on recognition of the highly variable external viral proteins and could be achieved with a single vaccine dose. The rAd vaccine was superior to the *ca* vaccine by certain measures, justifying continued investigation of this experimental vaccine even though *ca* vaccine is already available. This study highlights the potential for cross-protective vaccines as a public health option early in an influenza pandemic.

## Introduction

Influenza virus is a significant public health concern, with the average influenza season in the U.S. resulting in millions of cases and tens of thousands of deaths [1]. These deaths occur despite large-scale vaccination efforts, use of multiple antiviral influenza drugs, and in-patient care. Pandemic influenza represents an even greater concern. Current influenza vaccines function by targeting hemagglutinin (HA). Seasonal vaccines are not useful when a major antigenic change occurs in the circulating strain. Due to the time required for manufacture of new strain-matched vaccines, this can result in large proportions of the population being unprotected during the initial pandemic wave.

This situation is exemplified by the 2009 H1N1 pandemic. The 2009 pH1N1 is believed to have originated in Mexico during February of 2009 [2]. The virus soon spread to multiple countries, with the first U.S. case identified in mid-April 2009. The WHO officially declared an influenza pandemic in June. The time required for vaccine manufacture, testing and distribution delayed immunization in the U.S. until October, and initially restricted it to high risk individuals due to limited supply. By this time, infection rates were near peak levels. This delay occurred despite rapid identification of the novel strain.

The 2009 pH1N1 experience has highlighted the need to develop alternative vaccines operating by mechanisms of protection not dependent on antibodies against HA, the most variable influenza virus antigen. Instead, vaccination can target conserved antigens of influenza virus to generate heterosubtypic immunity protective against diverse influenza A virus strains and subtypes. While heterosubtypic immunity would not prevent infection, studies in animal models have clearly demonstrated that it can reduce the severity of illness and protect against lethal influenza virus challenge. Such cross-protective vaccines could be prepared and stockpiled prior to emergence of a pandemic virus, reducing the time between identification of a novel threat and deployment of the vaccine.

Various approaches to heterosubtypic vaccination against influenza have been studied, using one or more of the conserved viral proteins such as NP [3]–[6], matrix protein 1 (M1) or M2 [7]–[9], or the viral polymerases [5]. Delivery systems for such vaccines have utilized viral vectors [10], plasmid DNA [3], virus-like particles [11], proteins [12], or peptides [13]. Heterosubtypic immunity can be mediated by T cells and/or antibodies directed against such relatively conserved antigens such as NP, M1, M2 and the HA stem [7].

We have previously demonstrated that prime-boost immunization involving boosting with rAd expressing NP and M2 resulted in protection against challenge with divergent influenza strains, including virulent H1N1 and H3N2, and a highly pathogenic H5N1 avian virus [14], [15]. When the rAd vaccine was given intranasally without priming, protection was rapidly induced, with vaccinated animals protected from lethal challenge 2 weeks after a single immunization, and was long-lasting, with protective immunity still present 10 months after immunization [16].

While such vectored vaccines elicit potent immune responses focused against a limited range of viral antigens (such as NP and M2), to date they remain an experimental approach. An alternative may be to utilize live-attenuated, *ca* influenza vaccines, which are approved for human use and have been previously demonstrated to induce heterosubtypic immunity [14], [17], [18]. The *ca* and rAd-vectored vaccines can each be intranasally administered, thus providing the advantage of immunity at a relevant anatomic site.

In this study we have compared the effectiveness of NP+M2-rAd vaccine and a 1977 *ca* vaccine against infection with the 2009 pandemic virus. We describe results of testing a single mucosally-administered dose of these vaccines. We measured humoral and cellular immune responses elicited by the vaccines which were cross-reactive against pH1N1 antigens, as well as the ability of the vaccines to rapidly achieve protection against pH1N1 challenge. This study tests these vaccines against the recent pandemic H1N1 virus.

## Methods

### Ethics Statement

All animal protocols and procedures were approved by Institutional Animal Care and Use Committees at the Center for Biologics Evaluation and Research (protocol #1991-06) and the University of Georgia (protocol# A3437-01) in animal facilities accredited by the Association for Assessment and Accreditation of Laboratory Animal Care International. All experiments were performed according to institutional guidelines.

### Vaccines, viruses and peptides

Recombinant adenovirus vectors (Ad5-ΔE1ΔE3) expressing influenza A or B nucleoprotein (kindly provided by Gary Nabel, NIH Vaccine Research Center) and consensus M2 have been described previously [8], [19], and bulk stocks were produced by ViraQuest, Inc. (North Liberty, IA). The *ca* viruses A/Alaska/6/77 CR-29 clone 2 [H3N2], (A/Alaska *ca*), A/Hong Kong/123/77 CR-35 clone 2 [H1N1] (A/Hong Kong *ca*), and B/Ann Arbor/1/86 CRB-117, clone 19-2 (B/AA *ca*) were generously provided by Brian Murphy (NIAID, NIH, Bethesda, MD) and stocks were produced in eggs as described previously [20]. The 2009 pandemic H1N1 challenge virus is a mouse-adapted strain of A/California/04/09 (ma-CA/04) [21]. Challenge stocks were grown in MDCK cells for 48 hours at 37°C with 1 µg/ml TPCK-treated trypsin (Worthington Biochemical Inc.; Lakewood, NJ). HA protein from influenza virus A/California/04/09 (H1N1) was obtained through the NIH Biodefense and Emerging Infections Research Resources Repository, NIAID, NIH as a recombinant protein made in baculovirus, NR-15258. Recombinant NP was purchased from Imgenex (San Diego, CA) and has the A/PR/8/34 (PR8) sequence. HIV p24 gag_285–307_ and PR8 M2e were synthesized at the CBER core facility. All other PR8 and ma-CA/04 peptides were purchased from GenScript (Piscataway, NJ).

### Mice and immunizations

Six to eight week-old female BALB/cAnNCr (BALB/c) or C57BL/6NCr (B6) mice were purchased from NCI and maintained under specific pathogen-free conditions. Animals were anesthetized with isoflurane and immunized intranasally with 50 µl of vaccine dropwise to the nares. Animals received a dose of 5×10^9^ virus particles (vp) each of NP-rAd and M2-rAd, or 1×10^10^ vp of B/NP-rAd, or 1×10^5^ 50% tissue culture infectious doses (TCID_50_) each of A/Alaska *ca* and A/Hong Kong *ca*, or 2×10^5^ TCID_50_ of B/Ann Arbor *ca*.

### Challenge infections

Mice were anesthetized with 250 µl of Avertin (2-2-2-tribromoethanol, Aldrich Chemical Co.; Milwaukee, WI) administered via i.p. injection. Lethal doses (LD_50_) of ma-CA/04 were determined for each mouse strain. Challenge experiments used 5 LD_50_ of ma-CA/04 administered intranasally in 50 µl. Animals which lost ≥25% of their weight were humanely euthanized. Animals for analysis of lung viral titers were chosen before the start of the experiment. All challenge experiments were performed in ABSL-3 facilities at the University of Georgia.

### Antibody analysis

96-well flat bottom immunoplates (Nalge Nunc International; Rochester, NY) were coated at 4°C overnight with 1 µg/ml of recombinant NP (Imgenex; San Diego, CA) or 15 µg/ml of M2e peptide in 0.125 M saline, 0.007 M borate buffer. For HA ELISAs, recombinant H1 HA was diluted in PBS to 1 µg/ml for plate coating. ELISA assays were performed as previously described [22]. Optical densities (OD) were determined at 405 nm using a Multiskan EX spectrophotometer (Thermo Fisher Scientific Inc.; Waltham, MA) and Ascent analysis software. Data are expressed as endpoint titers, defined as the highest dilution of sample giving an OD>3 standard deviations (SD) above the mean of the starting dilution of naïve sera.

### ELISPOT analysis

Lungs tissue was harvested and processed and interferon-γ ELISPOT was performed as previously described [8], [15] by stimulation with indicated peptides.

### Determination of lung viral titers

For viral titer analyses, lungs were removed and placed into 2 ml Safe-Lock Tubes (Eppendorf AG; Hamburg, Germany) containing 1 ml of PBS and a 5 mm diameter steel ball-bearing. Lung tissue was homogenized using a TissueLyser (Qiagen; Valencia, CA) for 2 minutes at 30 oscillations/second. Lung homogenates were centrifuged at 2376 G for 5 minutes at 4°C and the clarified supernatant aliquoted and stored at −80°C until used. MDCK cells were plated in 96-well tissue culture plates at a density of 3×10^4^ cells per well (100 µl/well) the day before infection. Lung homogenate supernatants were diluted in a 10-fold series in quadruplicate using ‘Infection Media’ (MEM+2 mM L-glutamine+penicillin [100 IU/ml], streptomycin [100 µg/ml], amphotericin B [0.25 µg/ml]+TPCK-treated trypsin [1 µg/ml]). The cell monolayers were rinsed twice using PBS to remove all serum-containing medium, and then 200 µl of diluted test sample was added. Culture plates were then incubated at 37°C and 5% CO_2_ for 72 hours. After incubation, 50 µl of supernatant was removed for virus testing by hemagglutination. Fifty microliters of 0.5% chicken erythrocytes were added to supernatants in a 96-well round bottom plate and incubated for 30–60 minutes. Wells were observed for the presence of hemaggutination and TCID_50_ titers calculated using the Reed and Muench method [23].

### Graphs and statistics

All graphs were created using GraphPad Prism version 5 (GraphPad Software; La Jolla, CA). Statistics were performed using SigmaStat version 3.1 (Systat Software, Point Richmond, CA) or GraphPad Prism. Antibody and viral titer data were log-transformed prior to statistical analysis.

## Results

### Sequence differences between ma-CA/04 and the vaccines

Many studies of cross-protective vaccines have used a limited number of laboratory virus strains. Here we assess the potential of such vaccines against a recently emerged pandemic strain of direct relevance to human health. Effectiveness of cross-protective vaccination depends upon the impact of variation in key epitopes. While NP and M2 are relatively conserved among influenza A viruses, there are some potentially important differences between the sequences in the vaccines and the ma-CA/04 challenge virus. The dominant MHC class I NP epitopes recognized by BALB/c and B6 T cells are NP_147–155_ and NP_366–374_, respectively [24]–[26]. Table 1 compares the amino acid sequences of these and other epitopes tested in this study among the two vaccine sequences and the ma-CA/04 challenge virus. Table 2 lists the peptides used in this study. These peptides include segments of the NP and M2 proteins containing known or suspected MHC class I or MHC class II epitopes for BALB/c or B6 mice.

**Table 1:** Sequence variation in vaccine and challenge virus epitopes.

Epitope	Virus strain	Sequence	Accession # or Reference
NP_55–69_	A/PR/8/34	RLIQNSLTIERMVLS	AAM75159
NP_55–69_	ma-CA/04	RLIQNS**I**TIERMVLS	[21]
NP_55–69_	*A/AA/6/60	RLIQNSLTIERMVLS	AAA43451
NP_147–155_	A/PR/8/34	TYQRTRALV	AAM75159
NP_147–155_	ma-CA/04	TYQRTRALV	[21]
NP_147–155_	*A/AA/6/60	TYQRTRALV	AAA43451
NP_260–283_	A/PR/8/34	ARSALILRGSVAHKSCLPACVYGP	AAM75159
NP_260–283_	ma-CA/04	ARSALILRGSVAHKSCLPACVYG**L**	[21]
NP_260–283_	*A/AA/6/60	ARSALILRGSVAHKSCLPACVYGP	AAA43451
NP_366–374_	A/PR/8/34	ASNENMETM	AAM75159
NP_366–374_	ma-CA/04	ASNEN**VE**TM	[21]
NP_366–374_	*A/AA/6/60	ASNENMDTM	AAA43451
M2_2–24_	A/PR/8/34	SLLTEVETPIRNEWGCRCNGSSD	AAM75162
M2_2–24_	ma-CA/04	SLLTEVETP**T**R**S**EW**E**CRC**S**DSSD	[21]
M2_2–24_	*A/AA/6/60	SLLTEVETPIRNEWGCRCNDSSD	MFIV62

Sequence variations in known dominant NP T cell epitopes and M2e among influenza A viruses relevant to this study. Amino acids encoded by the challenge virus which differ from the A/PR/8/34 sequence are underlined. Amino acids encoded by the challenge virus which differ from the A/AA/6/60 sequence are bolded.

*A/Ann Arbor/6/60 (A/AA/6/60) is the backbone virus of the *ca* viruses used in this study.

**Table 2:** Peptides used in this study.

Virus^a^	Peptide^b^	Sequence	Analyses^c^
HIV	p24 gag_285–307_	QGPKEPFRDYVDRFYKTLRAEQA	B6+BALB/c
A/PR/8/34	NP_147–155_ d	TYQRTRALV	B6+BALB/c
A/PR/8/34	NP_55–69_	RLIQNSLTIERMVLS	BALB/c
ma-CA/04	NP_55–69_	RLIQNS**I**TIERMVLS	BALB/c
A/PR/8/34	NP_260–283_	ARSALILRGSVAHKSCLPACVYGP	B6
ma-CA/04	NP_260–283_	ARSALILRGSVAHKSCLPACVYG**L**	B6
A/PR/8/34	NP_366–374_	ASNENMETM	B6
ma-CA/04	NP_366–374_	ASNEN**VE**TM	B6
A/PR/8/34	M2_2–24_	SLLTEVETPIRNEWGCRCNGSSD	B6+BALB/c
ma-CA/04	M2_2–24_	SLLTEVETP**T**R**S**EW**E**CRC**S**DSSD	B6+BALB/c

NP and M2 peptides used in this study for analysis of immune responses. Amino acids in the challenge virus sequence which differ from the A/PR/8/34 sequence are underlined. Amino acids in the challenge virus sequence which differ from the A/Ann Arbor/6/60 sequence are bolded.

a Source virus of the specified peptide.

b Protein and sequence range of the peptide.

c Mice tested with the peptide.

d Sequence is 100% identical in vaccine and challenge viruses.

### Serum antibody levels following single dose vaccination

**Figure 1:**
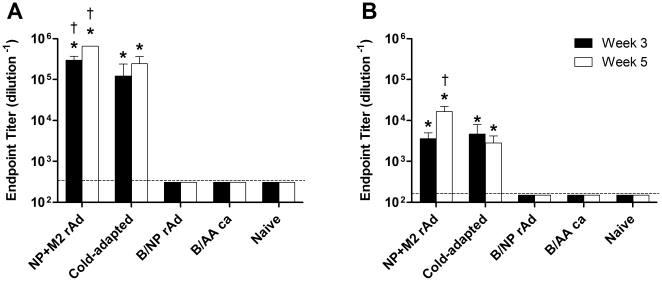
Anti-NP serum antibody titers. Anti-NP IgG levels in the serum of immunized mice at various timepoints following immunization. BALB/c (A) or B6 (B) mice were immunized with 1×10^10^ TCID_50_ of NP+M2-rAd or B/NP rAd, 2×10^5^ TCID_50_ of A/Alaska *ca*+A/Hong Kong *ca* (Cold-adapted) or B/AA *ca*, or left unvaccinated. Serum was obtained at 3 weeks (solid bars) or 5 weeks (open bars) post-vaccination and tested for anti-NP IgG as described in Materials and Methods. Shown are the mean endpoint titers ±SD; n = 5 per group. * P≤0.05 versus naïve, † P≤0.05 versus *ca*; One Way ANOVA with Student-Newman-Keuls post-test.

Mice were immunized once with rAd or *ca* viruses intranasally and bled 3 and 5 weeks after immunization. BALB/c mice immunized with NP+M2-rAd developed very high serum anti-NP IgG titers by week 3 post-immunization (Fig. 1A) and maintained a high level of antibody at week 5. Similarly, the *ca* vaccine elicited high anti-NP IgG titers in BALB/c mice (Fig. 1A). Control vaccines did not elicit detectable anti-NP IgG. The rAd and *ca* vaccines both induced anti-NP antibodies in B6 mice, albeit with lower titers (Fig. 1B).

**Figure 2:**
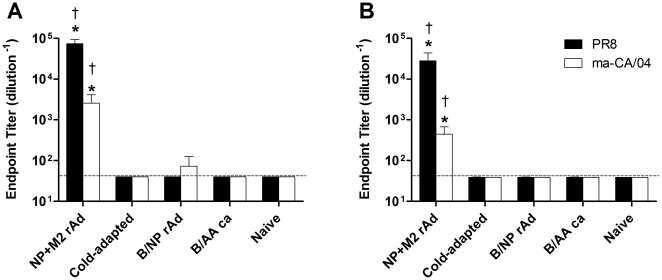
Anti-M2e serum antibody titers. Anti-M2e IgG levels in the serum of immunized mice at various timepoints following immunization. BALB/c (A and B) mice were immunized as in Figure 1 or left unvaccinated. Serum was obtained at 3 weeks (A) or 5 weeks (B) post-vaccination and tested for anti-M2e IgG antibody. Anti-M2e titers were determined using the PR8 peptide sequence (solid bars) and the ma-CA/04 peptide sequence (open bars). Shown are the mean endpoint titers ±SD; n = 5 per group. * P≤0.0001 versus naïve, † P≤0.05 versus *ca*; One Way ANOVA with Student-Newman-Keuls post-test.

BALB/c mice immunized with NP+M2-rAd developed a robust serum anti-M2e IgG response by 3 weeks post-immunization (Fig. 2A). We tested whether antibodies elicited against the PR8 M2e vaccine sequence recognize the divergent ma-CA/04 M2e sequence. Serum antibodies from immunized mice recognized M2e peptides from both PR8 and ma-CA/04. This robust anti-M2e response persisted through week 5 (Fig. 2B). Animals immunized with the *ca* vaccine or control vaccines failed to develop anti-M2e antibodies. When immunized with rAd and *ca* vaccines, B6 mice developed no detectable serum anti-M2e IgG at 3 weeks post-immunization (data not shown). Serum antibodies from mice immunized with the *ca* vaccine failed to cross-react with pandemic H1N1 HA above background levels (Fig. S1).

### Lung T cell responses following vaccination

**Figure 3:**
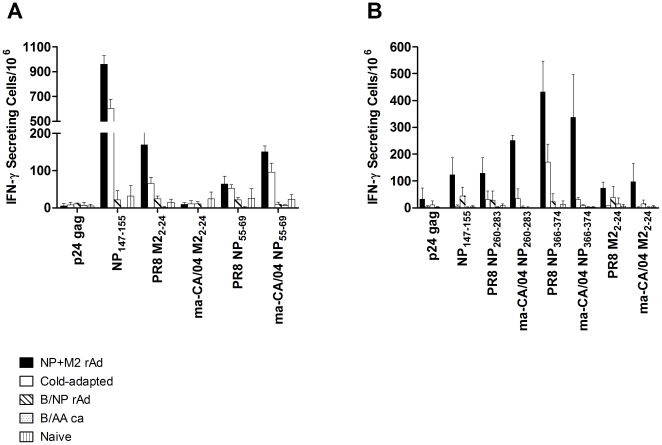
Lung T cell responses. IFN-γ responses of lung T cells following stimulation with peptides of vaccine sequences or challenge virus sequences. BALB/c (A) or B6 (B) mice were immunized as in Figure 1, or left unvaccinated. Three weeks after immunization, T cell responses were determined by IFN-γ ELISPOT as described in Materials and Methods. Pooled lung cells were tested using cells from 5 (BALB/c) or 7 (B6) mice per group. Shown are the mean of triplicate measurements of IFN-γ secreting cells per million ±SD.

Given that some T cell epitopes differ in sequence between the vaccine and the challenge virus, the T cell analysis was designed to address the impact of these differences. We examined T cell responses to NP and M2 of both the PR/8 and ma-CA/04 sequences using synthetic peptides containing previously reported dominant or sub-dominant T cell epitopes (Fig. 3). Testing was performed by IFN-γ ELISPOT assays on cells from lung tissue 3 weeks after immunization, the timepoint of challenge in the protection experiments.

As expected, BALB/c mice immunized with either NP+M2-rAd or *ca* vaccine developed a strong response to the NP_147–155_ peptide (Fig. 3A). These responses were stronger after immunization with NP+M2-rAd than *ca* virus. It is worth noting that the NP_147–155_ sequence is 100% conserved between the rAd, *ca*, and challenge virus strains. The T cell response to the PR8 M2_2–24_ sequence was moderate in lung cells from NP+M2-rAd-vaccinated mice, but quite low in the group immunized with *ca* vaccine. A response to the ma-CA/04 M2_2–24_ sequence was not detected in any of the BALB/c groups. While the MHC-II restricted T cell epitope in this region has not yet been fully defined, the PR8 and pH1N1 sequences differ by 5 residues, which seems to be sufficient to eliminate any cross-reactivity. T cell responses to the PR8 NP_55–69_ sequences overall were very low but marginally higher in response to the ma-CA/04 sequence.

Lung T cell responses were also examined for immunized B6 mice (Fig. 3B). The dominant NP MHC class I epitope recognized by B6 CD8+ T cells, NP_366–374_, induced a relatively strong response in NP+M2-rAd-immunized mice. The corresponding peptide of ma-CA/04 contains a single amino acid mutation at position 371 (M371V). It elicited a similar, albeit slightly lower, response despite the fact that the mutation is located in the T cell receptor (TCR) contact residue of the epitope. For mice immunized with *ca* vaccine, there was a moderate response to the PR8 NP_366–374_ peptide, but virtually none to the ma-CA/04 NP_366–374_ peptide. Moderate responses were observed with NP_260–283_ peptides. The responses to the two sequence variants were comparable, likely due to the fact that they differ by only one residue at the C-terminus. The response to the NP_260–283_ peptide was much greater with NP+M2-rAd vaccine than *ca* vaccine for either sequence variant.

### BALB/c and B6 mice are protected from challenge with ma-CA/04

**Figure 4:**
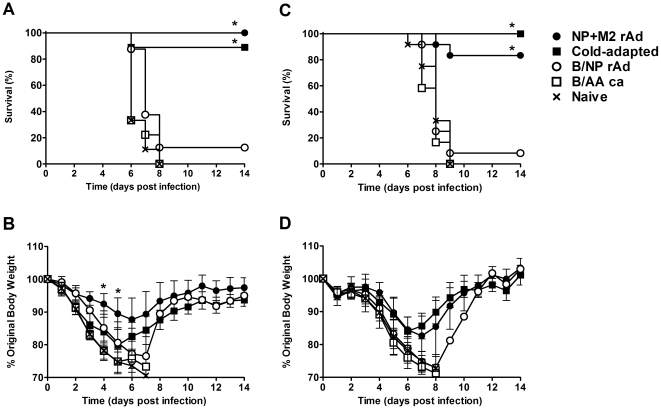
Weight loss and survival. Vaccine effectiveness in protection against challenge with ma-CA/04. Groups of BALB/c (A and B) or B6 (C and D) mice were immunized as in Figure 1, or left unvaccinated. Three weeks after immunization, animals were challenged with 5 MLD_50_ of ma-CA/04 intranasally and monitored for survival (A and C) and weight loss (B and D). Weight loss graph shows average of n = 9 (BALB/c) or n = 12 (B6) mice ±SD. * P≤0.0001 versus naïve; log rank test (A and C); or P≤0.01 versus naïve; two-way repeated measures ANOVA with Holm-Sidak post-test (B and D). No statistically significant differences were found between NP+M2-rAd and *ca* vaccine.

We next tested the ability of these partially cross-reactive immune responses to protect against a mouse-adapted 2009 H1N1 pandemic influenza virus (Fig. 4). Mice were challenged with ma-CA/04 three weeks after receiving a single dose of vaccine. All of the NP+M2-rAd-immunized BALB/c mice survived challenge (Fig. 4A) and experienced minimal weight loss (∼12% weight loss; Fig. 4B). Eight of the nine mice immunized with the *ca* vaccine survived challenge, but experienced considerably greater morbidity (∼20% weight loss). All control animals died between days 6 and 8 post-infection. For B6 mice, 10 out of 12 animals immunized with NP+M2-rAd and all animals in the *ca* vaccine group survived challenge (Fig. 4C). Both the NP+M2-rAd and *ca* groups lost weight at similar rates and with similar severities (∼15% weight loss; Fig. 4D). Surviving BALB/c and B6 mice immunized with the *ca* vaccine began gaining weight one day earlier than the NP+M2-rAd-immunized mice.

### Lung viral titers

**Figure 5:**
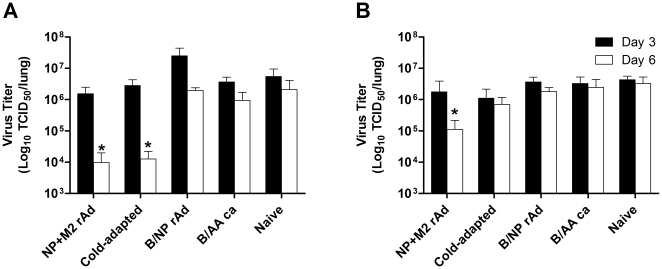
Lung viral titers. Viral replication in the lungs following ma-CA/04 challenge. BALB/c (A) or B6 (B) mice were immunized as in Figure 1, or left unvaccinated. Three weeks after immunization, animals were challenged with 5 MLD_50_ of ma-CA/04 intranasally. Lung viral titers were determined on day 3 (solid bars) and day 6 (open bars) by TCID_50_ assay as described in Material and Methods. Shown are the mean ±SD; n = 4 per group. * P≤0.05 versus naïve; Kruskal-Wallis test with Student-Newman-Keuls post test. No statistically significant differences were found between NP+M2-rAd and *ca* vaccine.

In order to monitor control of viral replication, lung viral titers were measured at days 3 and 6 following challenge (Fig. 5). In BALB/c mice, a single dose of NP+M2-rAd or *ca* virus reduced lung challenge virus titers by approximately 100-fold at day 6 post-challenge relative to naïve mice. No significant reduction in lung virus titers was observed at day 3 in NP+M2-rAd-immunized mice. In B6 mice, lung viral titers in the NP+M2-rAd group at day 6 were approximately 30-fold lower than in naïve mice (Fig. 5B). Animals in the *ca* vaccine group had lung viral titers slightly lower than naïve animals at day 6 but the difference was not statistically significant. No significant differences in lung titers were observed between any B6 groups at day 3 post-challenge. In either mouse strain, groups which received either of the control vaccines showed no significant difference from naïve mice at either time point.

## Discussion

This study examined cross-protection against pH1N1 by vaccines based on recombinant adenovirus and cold-adapted influenza viruses given intranasally. Both vaccines protected two strains of mice from lethal challenge with a mouse-adapted strain of the 2009 pandemic H1N1 virus. Protective immunity developed within three weeks of a single dose of each vaccine.

Although the cold-adapted vaccine we used in this study contained an H1N1 virus along with the heterosubtypic H3N2, we did not detect antibodies cross-reactive to the challenge virus HA (Fig. S1). This indicates that antibody-mediated neutralization was not responsible for protection. Serum antibody against M2e was also absent in *ca* immunized mice (Fig. 2). Possible mechanisms of cross-protection by *ca* vaccine include anti-NP antibodies present in the serum (Fig. 1), anti-NP cell-mediated immunity (Fig. 3), and cellular immunity to other components of the virus. Anti-NP antibodies have been shown to protect mice against challenge [27]–[29] as have T cells in the absence of antibody [30]. The relative contribution to protection of these different mechanisms is unknown.

In both C57BL/6 and BALB/c mice, we demonstrated that immunization with *ca* viruses can protect from pH1N1 challenge despite the mismatch between the HA and NA of the vaccine and challenge viruses. This contrasts with a recent study in which a seasonal commercial *ca* vaccine induced a significant NP-specific cellular response in ferrets, but failed to protect them from challenge with a 2009 pH1N1 isolate [31]. Both the ferret study and that reported here used the same *ca* vaccine backbone. While the dose of *ca* vaccine used in our experiments was clearly sufficient to protect mice, CTL responses in the lungs of mice immunized with *ca* virus have been shown to be highly dose dependent [32]. In the ferret experiment, the dose of *ca* vaccine recommended clinically for homologous protection was used, but it is unknown if a higher dose would be necessary for cross-protection. The discrepancy between the two studies may also be due to biological differences between mice and ferrets.

Antibodies against M2e are non-neutralizing, but have been shown to restrict virus growth in tissue culture and protect from influenza challenge *in vivo* [8], [11], [33], [34]. BALB/c mice developed high levels of serum antibodies against M2e following NP+M2-rAd but not *ca* vaccination. In confirmation of previous studies, B6 mice produced no M2e-specific antibodies with either immunization. However, the M2-rAd component was included because our previous studies have shown that immunization of B6 mice with NP+M2-rAd provides superior protection to NP-rAd alone, possibly due to a more robust T cell response [35]. While anti-M2e antibodies likely contribute to reduced morbidity in rAd-immunized BALB/c mice in our studies, they are not required for protection from lethality. Interestingly, others have reported induction of an M2e-specific antibody response by immunizing B6 mice with a chimpanzee rAd vector expressing a concatamer of 3 copies of M2e linked to NP as a fusion protein [9]. In that system, both anti-M2e antibodies and NP-specific CD8+ T cells are required for protection of B6 mice. The requirements for effective vaccination in B6 mice seem to vary depending on specific conditions of the experiment including immunization, vector, challenge strain and challenge dose.

For the conserved antigen NP, cross-reactive cellular immune responses have been shown to play an important role in protection [36]. The ma-CA/04 NP sequence is very similar (∼92% amino acid identity) to both the PR8 and A/Ann Arbor/6/60 NP sequences. A few residues where the sequences diverge occur in known T cell epitopes and the effect of those mutations on T cell cross-reactivity could be very important for cross-protection. NP_366–374_ is the dominant H2-D^b^-restricted epitope recognized by B6 mice. A single amino acid mutation at position 6 of this epitope (M371V) differentiates the PR8 sequence used in the NP+M2-rAd vaccine from the ma-CA/04 challenge sequence. Residues 5 and 9 are MHC anchor residues, while 4, 6, and 7 are TCR-contact residues [37], [38]. Homology between the PR8 and ma-CA/04 NP_366–374_ epitopes was sufficient for a substantial level of cross-reactivity in IFN-γ ELISPOT assays. Parallel T cell cross-reactivity has been demonstrated with respect to the M371V mutation in dual tetramer staining experiments [39].

A lesser degree of cross-reactivity to NP_366–374_ was observed for mice immunized with the *ca* vaccine. The NP from the *ca* vaccine is derived from A/Ann Arbor/6/60, the NP_366–374_ of which differs from the challenge sequence at both positions 6 (M371V) and 7 (D372E). Although these are conservative mutations with respect to the size and charge of the amino acids, two mutations in the TCR contact residues seem sufficient to impair cross-reactive recognition of the ma-CA/04 sequence by T cells. Dual tetramer staining experiments showed similar patterns [39].

The T cell responses to the dominant epitopes in both BALB/c and B6 mice were considerably greater following NP+M2-rAd than *ca* immunization. The lower response induced by *ca* vaccine could be due to differences in NP expression altering effective antigen dose, increased antigenic competition in *ca* virus, or augmentation of the immune response to rAd by adenovirus-mediated toll-like receptor activation. Interestingly, despite the lower T cell response, limited cross-reactivity to NP_366–374_, and lack of M2e-specific antibody in B6 mice, *ca* immunized mice were still protected from pH1N1 challenge.

There is considerable conservation of sequences identified as human T cell epitopes among H1 and H5 viruses and previous seasonal influenza strains. Studies have shown that human CD4 and CD8 T cells induced prior to the pandemic can cross-react with multiple pH1N1 antigens, especially the matrix, NP, and polymerase proteins [40], [41]. Additionally, pre-pandemic T cells recognizing conserved M1 epitopes can lyse pH1N1-infected target cells, as well as produce TNF-α and IFN-γ [42]. Similarly, multiple epitopes in internal antigens of H5N1 influenza viruses are recognized by T cells from individuals living in regions where these viruses have not circulated [43]. Given this level of pre-existing immunity in the adult human population, effective heterosubtypic vaccines may not need to induce *de novo* immune responses, but might merely need to boost pre-existing responses. Combined with our findings in this study, this provides support to the concept of a practical, fast-acting emergency vaccination regimen in the event of another pandemic.

In humans, prior immunity might alter effectiveness of a vaccine. Pre-existing antibodies to HA might in some cases interfere with replication of seasonal *ca* vaccine strains. However, some evidence suggests induction of CTL responses is still possible despite the presence of neutralizing antibody to the vaccine strain [44]. Pre-existing antibodies to adenovirus type 5 are common in humans, and there is concern that they may block use of rAd vectors. Possible methods to bypass pre-existing adenovirus immunity include use of nonhuman adenovirus vectors [9] or human adenoviruses of rare serotypes [45].

In this study we assessed protection at a relatively short interval (3 weeks) following a single immunization, to demonstrate the potential for emergency vaccination in response to a newly emerging pandemic. A recent study has demonstrated that *ca* virus is capable of protecting mice from pandemic virus 5 weeks after immunization [46]. Others have shown protection can be induced by *ca* vaccine as soon as 8 days following vaccination, albeit in a non-pandemic challenge model [18]. While both rAd and *ca* vaccines induced rapid T cell responses, the rAd vaccine was superior to *ca* vaccine by the criteria of IFN-γ secreting cells, morbidity following challenge in the case of BALB/c, and viral replication in the case of B6 mice. The better performance of the rAd vaccine justifies continued study of this experimental vaccine, even though *ca* vaccine is already available. We have shown previously that NP+M2-rAd immunization induces antibodies, T cell responses, and protection against highly virulent challenge viruses persisting for at least 10 months, but the duration of protection from the *ca* vaccine regimen used here is unknown.

The recent pH1N1 pandemic has highlighted the need for effective public health interventions when the next influenza pandemic emerges. The two candidate vaccines studied here represent althernative approaches to achieving heterosubtypic protection against pandemic influenza. Live attenuated influenza vaccines are already approved for clinical use in most age groups. While primarily intended to induce strain-matched immunity against HA, the data reported here and elsewhere [14], [17], [18] support the concept that they may also be valuable for inducing cross-protective immunity against the conserved antigens of influenza virus. Vectored vaccines (such as NP+M2-rAd) have been demonstrated to provide strong and durable protection in this and other animal studies [6], [9], [16], [47], [48], and some are in investigational clinical trials [7]. While still experimental, they were superior to *ca* vaccine by some measures in this study, and so merit further investigation. Cross-protective vaccines could be stockpiled prior to a pandemic and provide imperfect but valuable immunologic defense for many months, until a strain-matched vaccine could be manufactured and distributed in large quantities. If deployed early in the course of a pandemic, conserved antigen vaccines might reduce the human toll of morbidity and mortality, and lower the concomitant economic impact and burden on healthcare facilities and society.

## Supporting Information

Figure S1 **Immunization with** ***ca*** **does not induce cross-reactive antibodies to pH1N1 HA.** BALB/c and B6 mice (n = 5) were immunized with 2×10^5^ TCID_50_ of A/Alaska *ca*+A/Hong Kong *ca* or B/AA *ca* as in Figure 1 or left unvaccinated. Serum was obtained at 5 weeks post-vaccination from BALB/c (solid bars) and B6 (open bars) mice and tested for the presence of anti-pH1N1 HA IgG antibody. All serum samples were tested in the presence of control monoclonal antibodies (hatched bars). Shown are the mean endpoint titers ±SD; n = 5 per group. Positive control is H1N1 influenza A monoclonal antibody mixture from the 2009–2010 WHO influenza detection kit (anti-H1 mAb). Negative control is influenza B monoclonal antibody from the 2009–2010 WHO influenza detection kit (anti-FluB mAb).
(TIF)
