# Supplementary figures and images for: Cold-Adapted Influenza and Recombinant Adenovirus Vaccines Induce Cross-Protective Immunity against pH1N1 Challenge in Mice
Source: PLoS One. 2011 Jul 15;6(7):e21937. doi: 10.1371/journal.pone.0021937 (PMC3137593; doi:10.1371/journal.pone.0021937)

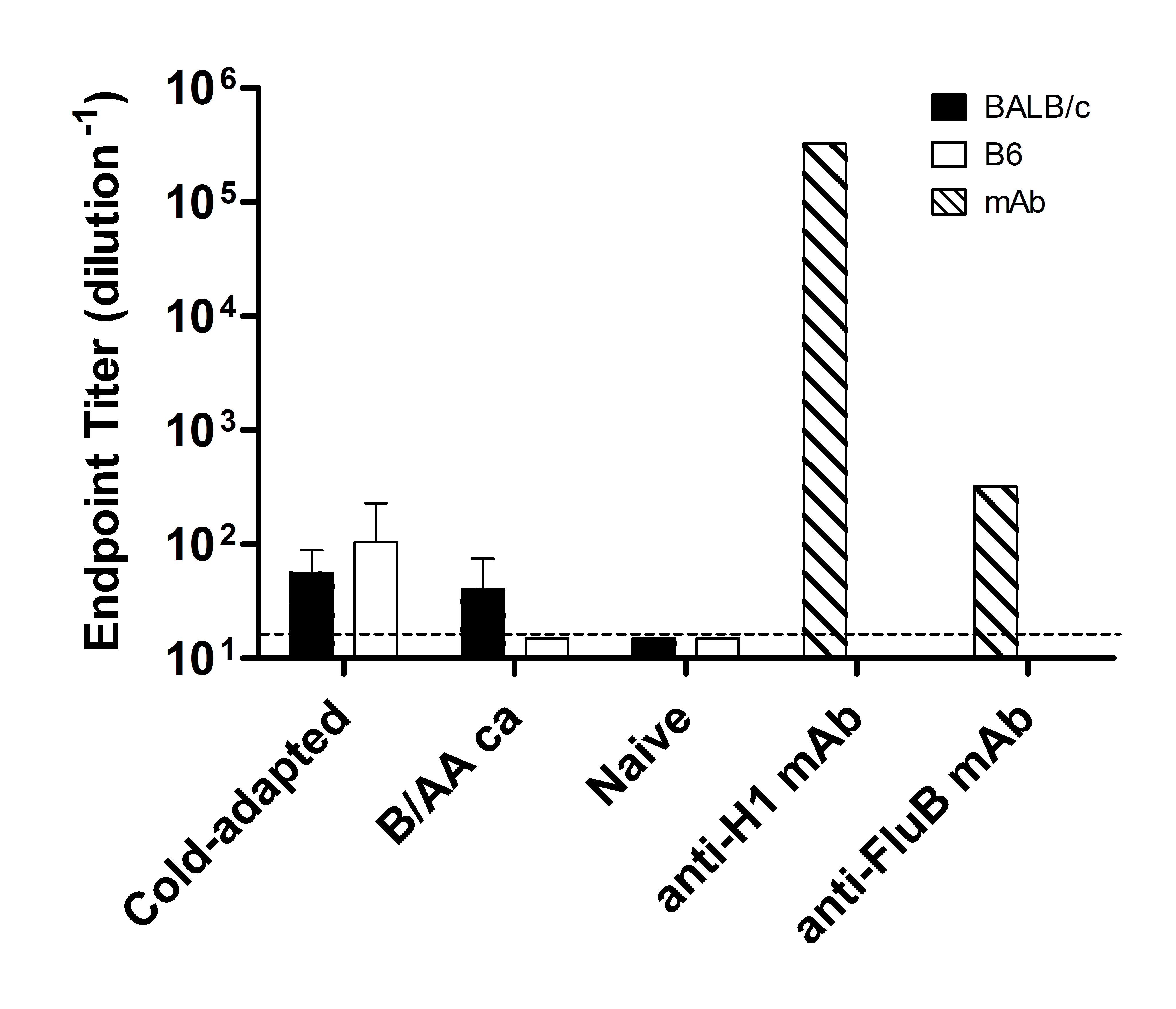

Supplement: Figure S1 — Immunization with ca does not induce cross-reactive antibodies to pH1N1 HA. BALB/c and B6 mice (n = 5) were immunized with 2×10^5^ TCID50 of A/Alaska ca+A/Hong Kong ca or B/AA ca as in Figure 1 or left unvaccinated. Serum was obtained at 5 weeks post-vaccination from BALB/c (solid bars) and B6 (open bars) mice and tested for the presence of anti-pH1N1 HA IgG antibody. All serum samples were tested in the presence of control monoclonal antibodies (hatched bars). Shown are the mean endpoint titers ±SD; n = 5 per group. Positive control is H1N1 influenza A monoclonal antibody mixture from the 2009–2010 WHO influenza detection kit (anti-H1 mAb). Negative control is influenza B monoclonal antibody from the 2009–2010 WHO influenza detection kit (anti-FluB mAb). (TIF) [file pone.0021937.s001.tif]
